# Experimental Oronasal Transmission of Chronic Wasting Disease Agent from White-Tailed Deer to Suffolk Sheep

**DOI:** 10.3201/eid2712.204978

**Published:** 2021-12

**Authors:** Eric D. Cassmann, S. Jo Moore, Justin J. Greenlee

**Affiliations:** US Department of Agriculture, Ames, Iowa, USA

**Keywords:** chronic wasting disease, CWD, chronic wasting disease agent, prions and related diseases, transmissible spongiform encephalopathies, animal diseases, experimental oronasal transmission, white-tailed deer, Suffolk sheep, zoonoses

## Abstract

Chronic wasting disease (CWD) is a fatal prion disease of cervids. We examined host range of CWD by oronasally inoculating Suffolk sheep with brain homogenate from a CWD-positive white-tailed deer. Sixty months after inoculation, 1/7 sheep had immunoreactivity against the misfolded form of prion protein in lymphoid tissue. Results were confirmed by mouse bioassay.

Transmissible spongiform encephalopathies (TSEs), also known as prion diseases, are a group of fatal neurologic diseases caused by a misfolded form of the prion protein (PrP^Sc^). Several TSEs affect livestock, including scrapie in sheep and chronic wasting disease (CWD) in cervids.

Susceptibility of sheep to the agent of scrapie is determined by the host prion protein genotype. Three polymorphisms at codons 136, 154, and 171 of the prion protein gene occur in sheep. The haplotype A_136_R_154_R_171_ is associated with resistance to scrapie, whereas VRQ is linked with susceptibility. Likewise, the deer prion protein genotype GG_96_ is overrepresented in cases of CWD.

CWD was identified in captive mule deer in Colorado, USA, in 1967 (*1*). Since then, CWD has been reported in >24 states in the United States, 2 provinces in Canada, and South Korea (*2*,*3*). During 2016, CWD was reported in Europe, and it has since been detected in 3 Nordic countries (Norway, Sweden, and Finland), although CWD strains in Europe were recently shown to be distinct from strains in North America (*4*). Because of human consumption of cervid meat products and intermingling of various livestock species with wild cervid populations, there is major interest in characterizing the possible host range of CWD.

Scrapie has been implicated as the possible source of CWD in cervids (*5*). This finding is supported by in vitro conversion of sheep prion protein by infectious CWD prions (*6*) and glycoprofile similarities between scrapie and CWD prions (*7*). Another similarity between scrapie and CWD is prominent lymphoid accumulation of PrP^Sc^ in both species affected (*5*). Experimental transmission of mule deer CWD to Suffolk sheep by intracranial inoculation, a highly artificial route of transmission, has been performed (*8*). Widespread peripheral lymphoid accumulation of PrP^Sc^ is retained in intracranially CWD inoculated sheep.

The objective of this study was to test the oronasal susceptibility of sheep to the agent of CWD. We report the preliminary findings of an ongoing multiyear study.

## The Study

Initially, we oronasally inoculated (*9*) seven Suffolk lambs (3‒4 months of age) with the V_136_R_154_Q_171_/ARQ (n = 2), ARQ/ARQ (n = 4), or ARQ/ARR (n = 1) prion protein genotype and 0.1 g of 10% (wt/vol) brain homogenate from a GG_96_ white-tailed deer that had CWD. The sheep were housed indoors in a Biosafety Level 2 agriculture facility separate from scrapie-affected sheep. At 60 months postinoculation, the initial experimental endpoint, sheep were asymptomatic, and all 7 sheep were culled.

We performed a postmortem examination on each sheep and collected a full spectrum of tissues, which we froze and stored in 10% neutral-buffered formalin. To evaluate lymphoinvasion and neuroinvasion, we tested tissues from the brainstem at the obex and pons, third eyelid, palatine tonsil, lymph nodes (mesenteric and retropharyngeal), spleen, and ileum. We processed the formalin-fixed tissues, embedded in paraffin, and sectioned at optimal thickness (brain, 4 µm; lymphoid, 3 µm; and other tissues, 5 µm) for subsequent staining with hematoxylin and eosin and immunohistochemical (IHC) analysis. We used a cocktail of PrP^Sc^ monoclonal antibodies (F89/160.1.5 and F99/97.6.1; 5 μg/mL) for IHC.

**Figure 1:**
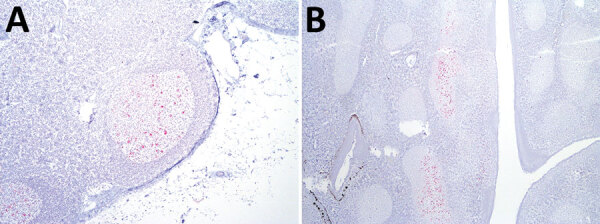
Immunoreactivity against misfolded form of the prion protein (red) in lymphoid tissue from a sheep oronasally inoculated with the agent of chronic wasting disease from white-tailed deer. A) Retropharyngeal lymph node (original magnification ×100.) B) Palatine tonsil (original magnification ×40). We used a cocktail of monoclonal antibodies (F89/160.1.5 and F99/97.6.1).

Examination of IHC-stained tissues showed PrP^Sc^ in the retropharyngeal lymph node (Figure 1, panel A) and palatine tonsil (Figure 1, panel B) of 1 sheep inoculated with the ARQ/ARQ genotype. The retropharyngeal lymph node was also positive by enzyme immunoassay (EIA) (HerdChek; IDEXX Laboratories, https://www.idexx.com) at initial (optical density 0.99; negative cutoff value 0.186) and repeat (optical density 0.559; negative cutoff value 0.178) tests. The palatine tonsil was negative by EIA.

To confirm prion disease infectivity in the retropharyngeal lymph node, we performed bioassays in Tg12 cervidized (*10*) and Tg338 ovinized (*11*) transgenic mice. Mice expressed the transgene for the elk prion protein polymorphism MM_132_ (Tg12) and the ovine prion protein polymorphisms V_136_R_154_Q_171_ (Tg338). We homogenized fresh frozen lymph nodes to 10% (wt/vol) and enriched them by repeated rounds of differential centrifugation; we intracranially inoculated mice with 20 μL of 10% (wt/vol) equivalent enriched homogenate. The Tg12 bioassay had a partial attack rate of 5/9 mice. Most (4/5) dead Tg12 mice were strongly positive by EIA (optical density 4.0) and had an average incubation period of 511 days.

**Figure 2:**
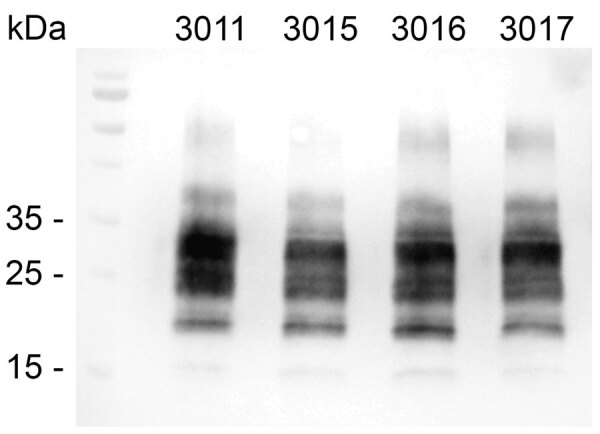
Western blot analysis showing proteinase K‒resistant misfolded form of the prion protein (PrP^Sc^) in brains of 4 Tg12 mice. Mice were intracranially inoculated with a homogenate made from retropharyngeal lymph node of a sheep oronasally inoculated with the agent of chronic wasting disease. Tg12 brain was prepared as a 10% (wt/vol) homogenate with phosphate-buffered saline. A total of 1 mg of tissue equivalent was treated with proteinase K (90 μg/mL) before electrophoresis. Immunodetection of PrP^Sc^ was performed overnight at 4°C with monoclonal antibody Sha31 (dilution 1:10,000). Left lane, molecular mass ladder. kDa, kilodaltons.

Western blots of these 4 Tg12 mice confirmed the presence of proteinase K‒resistant PrP^Sc^ in the brains (Figure 2). The positive EIA results were obtained from brain homogenates in Tg12 mice; the spleens were negative for PrP^Sc^. For the Tg338 bioassay (n = 15), brains and spleens were negative by EIA. Four Tg338 mice that died or were euthanized because of intercurrent disease at 254, 462, 629, and 657 days postinoculation were negative by EIA. The rest of the Tg338 mice were negative at the study endpoint, 700 days postinoculation.

## Conclusions

The oronasal susceptibility of sheep to the agent of CWD is a major finding in light of its possible effect on risk assessment and understanding possible transmission of CWD to noncervid species in field conditions. Interspecies transmission of TSEs is less likely when the experimental species barrier between hosts is strong (*12*). One study demonstrated that the CWD agent does not readily transmit to transgenic ovinized mice (*13*). However, another study reported lifelong replication of PrP^Sc^ in the spleen after intracranial inoculation of the CWD agent in Tg338 ovinized mice (*14*). The finding of extraneuronal PrP^Sc^ in 1 sheep 5 years after oral inoculation suggests that sheep are unlikely to develop neurologic disease after natural exposure to the agent of CWD, but they might serve as asymptomatic carriers under the right conditions.

In this study, we used a relatively low dose (0.1 g) of brain homogenate. These results are intriguing, but they do not assess potential modes of transmission that could occur in the field, such as nose-to-nose contact or environmental contamination. In our ongoing multiyear study, 1 sheep had PrP^Sc^-positive lymphoid tissue but no evidence of neuroinvasion 5 years postinoculation. This time interval is an extremely protracted incubation period. Had we continued this experiment, it is unknown how long the sheep would have remained asymptomatic or whether they would have eventually developed clinical disease. Because PrP^Sc^ was detected in lymphoid tissues of the head, the possibility that this sheep might have been shedding infectivity into the environment cannot be ruled out.

Positive bioassay results in Tg12 mice confirm CWD infectivity in the lymph node. Negative results in Tg338 mice could be explained by a donor/host mismatch between the ARQ donor sheep and VRQ expressing mice. Pursuing bioassays in A_136_-expressing transgenic mice could be more fruitful.

Interspecies transmission events might increase the pathogenicity of an infectious prion on subsequent transmission to other species (*15*). Thus, exploration of potential new host ranges of this CWD isolate and performing human health risk assessments will provide useful information for this prion.

## References

[1] Williams ES, Young S. Spongiform encephalopathies in Cervidae. Rev Sci Tech. 1992;11:551–67. 10.20506/rst.11.2.611 1617203

[2] Carlson C, Hopkins C, Nguyen N, Richards B, Walsh D, Walter WD. Chronic wasting disease: status, science, and management support by the US Geological Survey. US Geological Survey; March 2018. Report no. Open-File Report 2017–1138 [cited 2021 Aug 23]. https://pubs.er.usgs.gov

[3] Haley NJ, Hoover EA. Chronic wasting disease of cervids: current knowledge and future perspectives. Annu Rev Anim Biosci. 2015;3:305–25. 10.1146/annurev-animal-022114-111001 25387112

[4] Nonno R, Di Bari MA, Pirisinu L, D’Agostino C, Vanni I, Chiappini B, et al. Studies in bank voles reveal strain differences between chronic wasting disease prions from Norway and North America. Proc Natl Acad Sci U S A. 2020;117:31417–26. 10.1073/pnas.2013237117 33229531 PMC7733848

[5] Williams ES. Chronic wasting disease. Vet Pathol. 2005;42:530–49. 10.1354/vp.42-5-530 16145200

[6] Raymond GJ, Bossers A, Raymond LD, O’Rourke KI, McHolland LE, Bryant PK III, et al. Evidence of a molecular barrier limiting susceptibility of humans, cattle and sheep to chronic wasting disease. EMBO J. 2000;19:4425–30. 10.1093/emboj/19.17.4425 10970836 PMC302048

[7] Race RE, Raines A, Baron TG, Miller MW, Jenny A, Williams ES. Comparison of abnormal prion protein glycoform patterns from transmissible spongiform encephalopathy agent-infected deer, elk, sheep, and cattle. J Virol. 2002;76:12365–8. 10.1128/JVI.76.23.12365-12368.2002 12414979 PMC136873

[8] Hamir AN, Kunkle RA, Cutlip RC, Miller JM, Williams ES, Richt JA. Transmission of chronic wasting disease of mule deer to Suffolk sheep following intracerebral inoculation. J Vet Diagn Invest. 2006;18:558–65. 10.1177/104063870601800606 17121083

[9] Moore SJ, Smith JD, Greenlee MHW, Nicholson EM, Richt JA, Greenlee JJ. Comparison of two US sheep scrapie isolates supports identification as separate strains. Vet Pathol. 2016;53:1187–96. 10.1177/0300985816629712 26936223

[10] Kong Q, Huang S, Zou W, Vanegas D, Wang M, Wu D, et al. Chronic wasting disease of elk: transmissibility to humans examined by transgenic mouse models. J Neurosci. 2005;25:7944–9. 10.1523/JNEUROSCI.2467-05.2005 16135751 PMC6725448

[11] Vilotte JL, Soulier S, Essalmani R, Stinnakre MG, Vaiman D, Lepourry L, et al. Markedly increased susceptibility to natural sheep scrapie of transgenic mice expressing ovine prp. J Virol. 2001;75:5977–84. 10.1128/JVI.75.13.5977-5984.2001 11390599 PMC114313

[12] Torres JM, Espinosa JC, Aguilar-Calvo P, Herva ME, Relaño-Ginés A, Villa-Diaz A, et al. Elements modulating the prion species barrier and its passage consequences. PLoS One. 2014;9:e89722. 10.1371/journal.pone.0089722 24608126 PMC3946430

[13] Tamgüney G, Giles K, Bouzamondo-Bernstein E, Bosque PJ, Miller MW, Safar J, et al. Transmission of elk and deer prions to transgenic mice. J Virol. 2006;80:9104–14. 10.1128/JVI.00098-06 16940522 PMC1563923

[14] Béringue V, Herzog L, Jaumain E, Reine F, Sibille P, Le Dur A, et al. Facilitated cross-species transmission of prions in extraneural tissue. Science. 2012;335:472–5. 10.1126/science.1215659 22282814

[15] Espinosa JC, Andréoletti O, Castilla J, Herva ME, Morales M, Alamillo E, et al. Sheep-passaged bovine spongiform encephalopathy agent exhibits altered pathobiological properties in bovine-PrP transgenic mice. J Virol. 2007;81:835–43. 10.1128/JVI.01356-06 17079295 PMC1797487

